# Base of coracoid process fracture with acromioclavicular dislocation in a child

**DOI:** 10.1186/1749-799X-5-77

**Published:** 2010-10-18

**Authors:** Prithee Jettoo, Gavin de Kiewiet, Simon England

**Affiliations:** 1 Department of Trauma and Orthopaedics, Sunderland Royal Hospital, Sunderland SR4 7TP, UK

## Abstract

Fracture of the coracoid process is a rare injury. It can be easily missed when associated with other injuries to the shoulder girdle, for instance, acromioclavicular joint (ACJ) dislocation. Clinical attention is easily drawn to the more obvious ACJ dislocation, hence, the need for further radiological evaluation. We report an unusual case of fracture of the base of coracoid process associated with a true acromioclavicular joint dislocation in a 12 year old boy, with no separation of the epiphyseal plate, as one might expect. Treatment also remains controversial. Our patient underwent open reduction internal fixation of the acromioclavicular joint and coracoid process. He subsequently made an uneventful progress with pain free full range of shoulder movement at 5 months, and was discharged at 9 months.

## Introduction

Coracoid fracture is an uncommon injury, accounting for only 2% to 13% of all scapular fractures and approximately 1% of all fractures [1-3]. Acromioclavicular joint dislocation is a very rare injury in a child below the age of thirteen [4]. We report an interesting case of fracture of the coracoid process associated with acromioclavicular joint dislocation in a child. He underwent open reduction internal fixation of the acromioclavicular joint and coracoid process. He subsequently made a good progress with pain free full range of shoulder movement.

## Case presentation

A twelve year old boy came off a rope swing from four metres, landed on his right shoulder and sustained an isolated injury to his right shoulder girdle. He complained of pain and swelling. Clinically, he had a prominent lateral clavicle associated with swelling, marked bruising and tenderness over his right shoulder and scapular area. His range of motion was restricted. He had no evidence of a brachial plexus injury, and had no vascular compromise.

**Figure 1:**
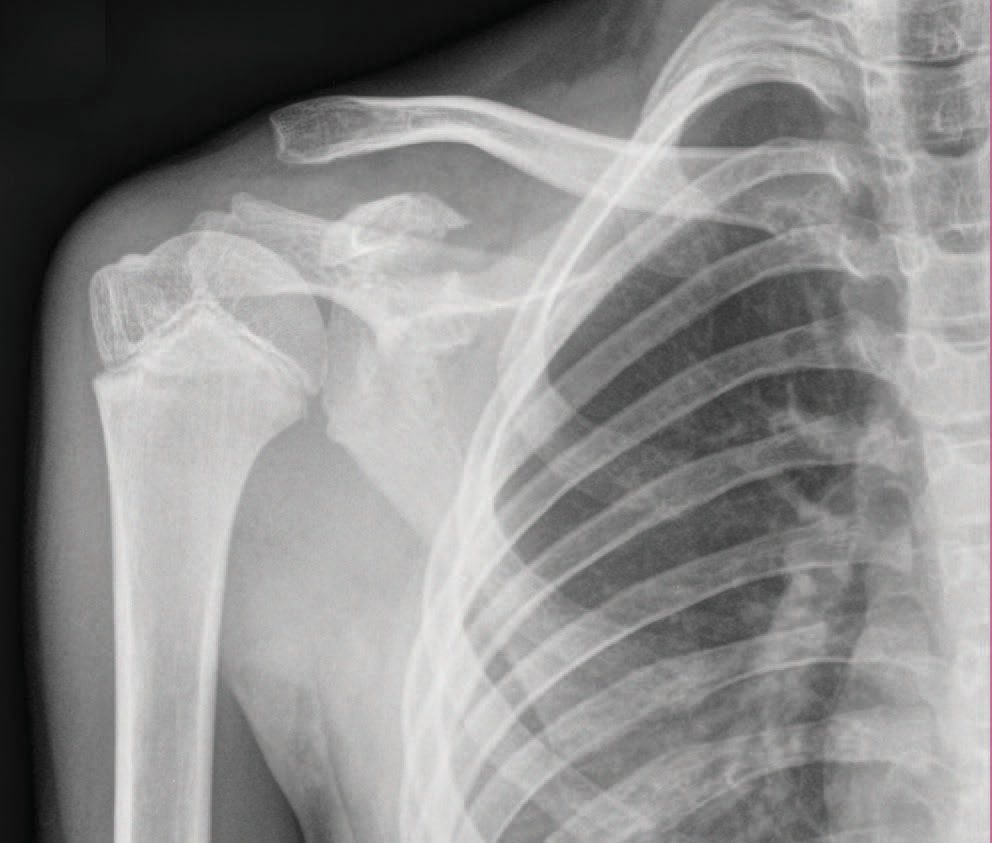
**Radiograph showing a standard anteroposterior view of the right shoulder with dislocation of the acromioclavicular joint and fracture of base of coracoid process**.

**Figure 2:**
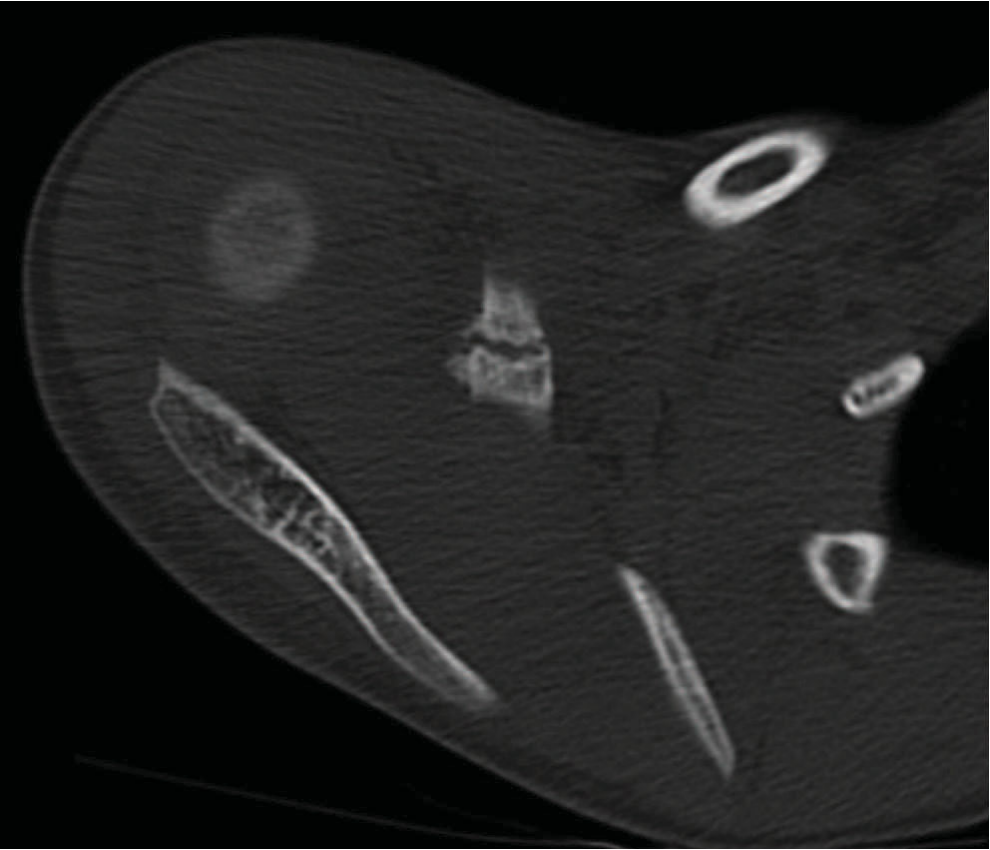
**Axial CT image of the right shoulder with an intact epiphyseal plate of the coracoid process**.

**Figure 3:**
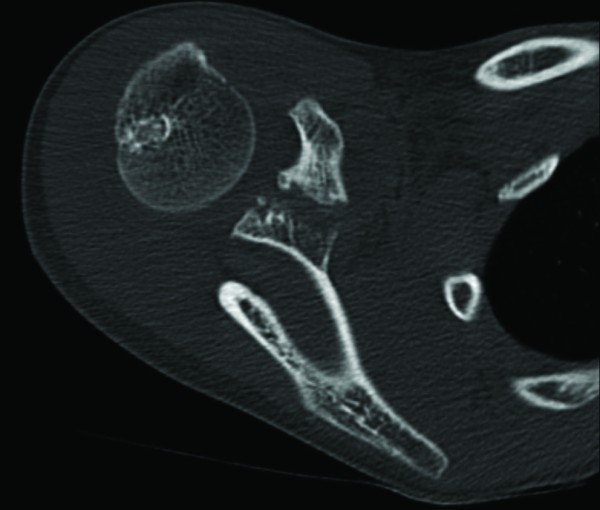
**Axial CT image with a fracture of the base of coracoid process**.

**Figure 4:**
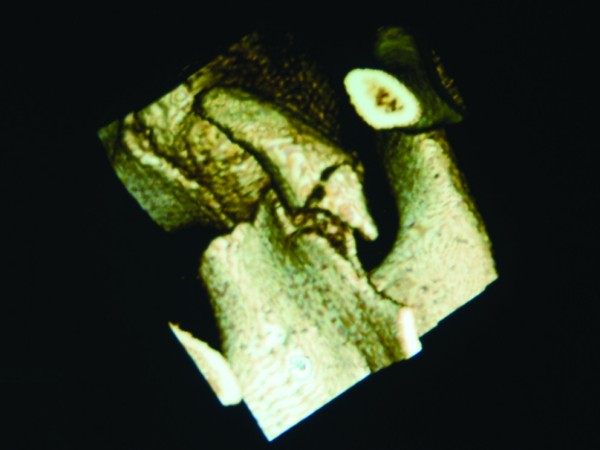
**Three-dimensional reconstructions of the CT scan give a spatial view of the coracoid fracture fragment**.

**Figure 5:**
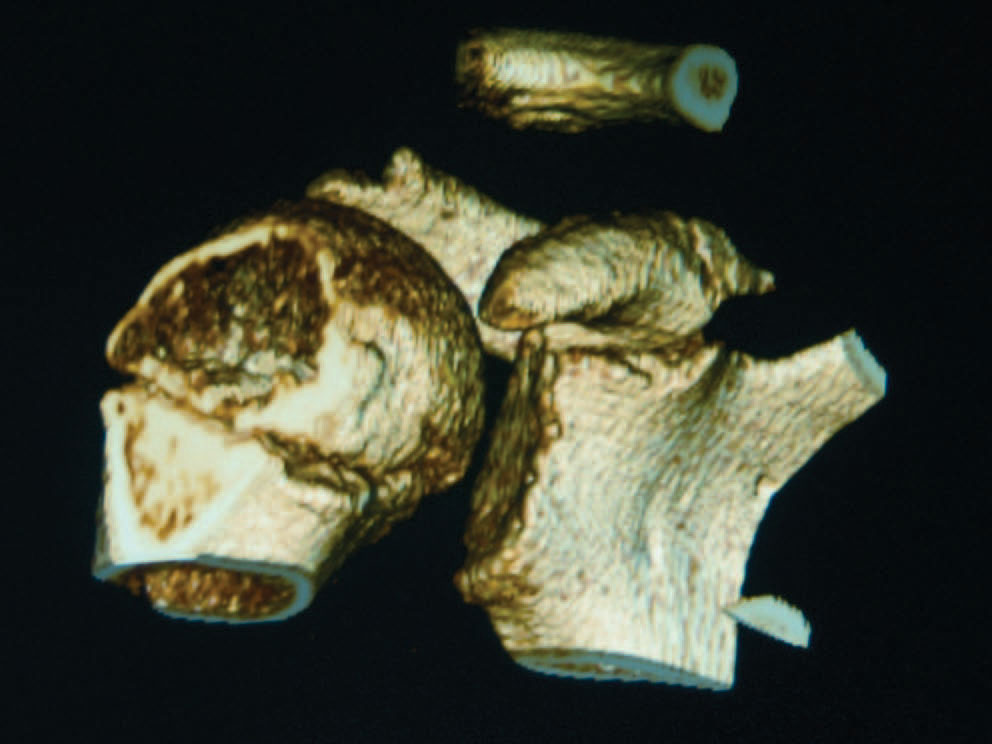
**Three-dimensional reconstructions of the CT scan show a base of coracoid fracture with an intact epiphyseal plate**.

His initial radiographs showed a widely displaced acromioclavicular joint with possible coracoid process fracture (Figure 1). He had a computed tomography (CT) scan, which confirmed the associated fracture at the base of his coracoid process (Figures 2, 3). A three dimensional CT scan reconstruction showed a spatial view of the coracoid process fragment (Figures 4, 5)

**Figure 6:**
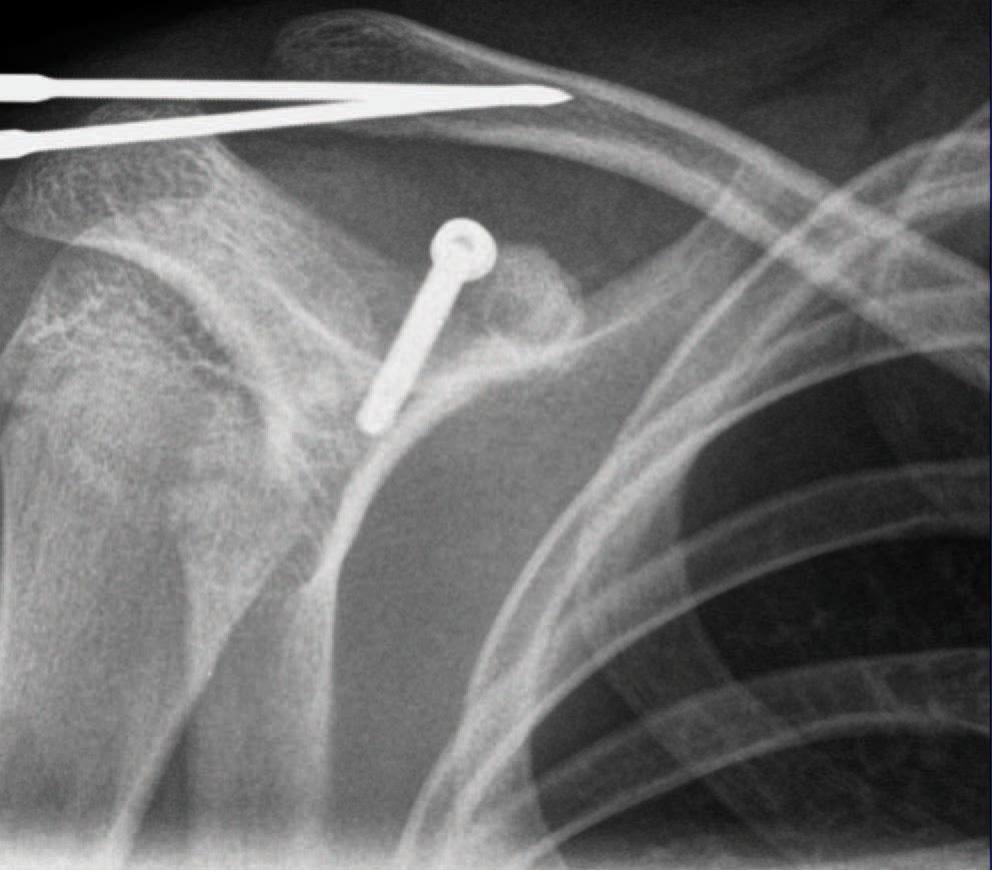
**Post-operative radiograph anteroposterior of the right shoulder**.

**Figure 7:**
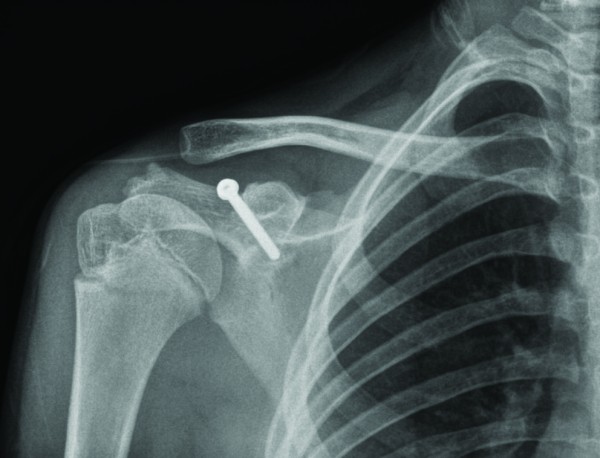
**Post-operative radiograph after removal of threaded pins, with reduction of acromioclavicular joint maintained**.

He underwent surgical intervention with reduction and fixation of the acromioclavicular joint with two threaded half pins and screw fixation of the base of coracoid fracture (Figure 6). Intraoperatively, his coracoclavicular and coracoacromial ligaments were intact and attached to the fracture fragment; but he had a disrupted acromioclavicular capsule. Post-operatively, a shoulder immobiliser was applied; and he started intermittent graded right shoulder movement. The threaded pins were removed four weeks later (Figure 7). At 3 months follow-up, the patient had a good range of movement of his right shoulder, with occasional clicking on abduction. He was advised to continue with shoulder exercises and avoid strenuous activity. His radiograph showed that position was maintained. At 5 months, he had full active pain free range of movement with resolution of clicking on abduction of his right shoulder. At 9 months follow-up, he had gone to normal activities, and was discharged from clinic.

## Discussion

An isolated coracoid fracture can occur by direct trauma to the shoulder girdle. It is suggested that an avulsion fracture of the coracoid could be caused by the sudden and violent contraction of the conjoined tendon [5] of the short head of the biceps, coracobrachialis and pectoralis minor or by the acromioclavicular ligaments. The latter mechanism is believed to account for fracture patterns seen in children.

A coracoid fracture can be isolated or associated with an injury complex, including any of acromiclavicular disruption, clavicular fracture, acromial fracture, scapular spine fracture or glenoid fracture [2,3].

Fracture sites described in adults are the base of the process, including the upper region of the glenoid, the middle portion and the tip.

The coracoid is thought to have two main ossification centres, one at the base of the process, and an accessory ossification centre at its tip [6]. Avulsion injuries in children result in fracture at the epiphyseal base of the coracoid base and the upper quarter of the glenoid or through the tip of the coracoid process [7].

Epiphyseal separation of the coracoid process with concomitant acromioclavicular sprain has also been reported in adolescents [6]. In the developing skeleton, the epiphyseal plate is weaker than the coracoclavicular ligaments. Interestingly, we describe a rare injury in this twelve year old boy with an avulsion fracture of base of coracoid with acromioclavicular dislocation. There was no epiphyseal plate separation, as one might expect in this age group (Figures 5 &6), but the base of the coracoid was avulsed, an injury usually seen in patients in the second or third decade of life [6]. Intra-operatively, we found intact coracoclavicular (conoid and trapezoid) and corocoacromial ligaments, which reflects the elasticity and resiliency of the ligaments in the younger child, but there was disruption of the acromioclavicular joint capsule.

The treatment of this type of injury is rather controversial. Both operative and non-operative treatment methods [7-9] have been reported. In an injury complex, involving small bony avulsion fracture of the angle of the coracoid process, some adopt a treatment principle similar to that developed for grade III acromioclavicular joint disruptions [10]. In this child, we opted for surgical intervention to allow early postoperative rehabilitation with mobilisation exercises. We proceeded with open reduction and internal fixation of both sites with this displaced base of coracoid fracture to avoid the adverse long-term effects of an acromioclavicular dislocation and a non union of the coracoid process.

Albeit rare, a coracoid process fracture is an injury that can be missed, when combined with an acromioclavicular joint dislocation. Clinical attention is easily drawn to the more obvious ACJ dislocation, hence, the need for further radiological evaluation. We seek to draw attention to this rare injury complex in a twelve year old, and present the good outcome with surgical intervention.

## List of abbreviations

ACJ: acromioclavicular joint

## Consent

Written informed consent was obtained from the patient for publication of this case and any accompanying images. A copy of the written consent is available for review by the Editor-in-Chief of this journal.

## Competing interests

The authors declare that they have no competing interests.

## Authors' contributions

PJ conceived the idea and co-wrote the paper. GdeK performed the surgery and contributed to the discussion. SE assisted with the radiology and contributed to the discussion. All authors have read and approved the final manuscript.
